# Impact of Aging, Cytomegalovirus Infection, and Long-Term Treatment for Human Immunodeficiency Virus on CD8^+^ T-Cell Subsets

**DOI:** 10.3389/fimmu.2018.00572

**Published:** 2018-03-21

**Authors:** Ellen Veel, Liset Westera, Rogier van Gent, Louis Bont, Sigrid Otto, Bram Ruijsink, Huib H. Rabouw, Tania Mudrikova, Annemarie Wensing, Andy I. M. Hoepelman, José A. M. Borghans, Kiki Tesselaar

**Affiliations:** ^1^Laboratory of Translational Immunology, University Medical Center Utrecht, Utrecht, Netherlands; ^2^Department of Internal Medicine and Infectious Diseases, University Medical Center Utrecht, Utrecht, Netherlands; ^3^Department of Medical Microbiology, University Medical Center Utrecht, Utrecht, Netherlands

**Keywords:** healthy aging, cytomegalovirus, human immunodeficiency virus infection, combination antiretroviral treatment, CD8^+^ T-cells

## Abstract

Both healthy aging and human immunodeficiency virus (HIV) infection lead to a progressive decline in naive CD8^+^ T-cell numbers and expansion of the CD8^+^ T-cell memory and effector compartments. HIV infection is therefore often considered a condition of premature aging. Total CD8^+^ T-cell numbers of HIV-infected individuals typically stay increased even after long-term (LT) combination antiretroviral treatment (cART), which is associated with an increased risk of non-AIDS morbidity and mortality. The causes of these persistent changes in the CD8^+^ T-cell pool remain debated. Here, we studied the impact of age, CMV infection, and LT successful cART on absolute cell numbers in different CD8^+^ T-cell subsets. While naïve CD8^+^ T-cell numbers in cART-treated individuals (*N* = 38) increased to healthy levels, central memory (CM), effector memory (EM), and effector CD8^+^ T-cell numbers remained higher than in (unselected) age-matched healthy controls (*N* = 107). Longitudinal analysis in a subset of patients showed that cART did result in a loss of memory CD8^+^ T-cells, mainly during the first year of cART, after which memory cell numbers remained relatively stable. As CMV infection is known to increase CD8^+^ T-cell numbers in healthy individuals, we studied whether any of the persistent changes in the CD8^+^ T-cell pools of cART-treated patients could be a direct reflection of the high CMV prevalence among HIV-infected individuals. We found that EM and effector CD8^+^ T-cell numbers in CMV^+^ healthy individuals (*N* = 87) were significantly higher than in CMV^−^ (*N* = 170) healthy individuals. As a result, EM and effector CD8^+^ T-cell numbers in successfully cART-treated HIV-infected individuals did not deviate significantly from those of age-matched CMV^+^ healthy controls (*N* = 39). By contrast, CM T-cell numbers were quite similar in CMV^+^ and CMV^−^ healthy individuals across all ages. The LT expansion of the CM CD8^+^ T-cell pool in cART-treated individuals could thus not be attributed directly to CMV and was also not related to residual HIV RNA or to the presence of HIV-specific CM T-cells. It remains to be investigated why the CM CD8^+^ T-cell subset shows seemingly irreversible changes despite years of effective treatment.

## Introduction

Infection with human immunodeficiency virus (HIV) leads to substantial changes in the T-cell compartment. Not only the CD4^+^ T-cell compartment—the decline of which forms one of the main characteristics of HIV infection—but also the CD8^+^ T-cell pool undergoes significant changes in HIV infection. There is a relative abundance of highly differentiated T-cells, characterized by a reduced capacity to proliferate, short telomere lengths (1) and changes in cytokine secretion capacity (2, 3). A progressive decline in naïve CD8^+^ T-cell numbers occurs concomitant with an increase in memory CD8^+^ T-cell numbers (4). Since these changes are reminiscent of the changes in the T-cell compartment observed during healthy aging (5, 6), HIV infection is often regarded a condition of premature immunological aging (7). While immune senescence during healthy aging is thought to result from the multiple rounds of activation of the immune system throughout life, the chronic immune activation induced by HIV may accelerate this aging process (8, 9).

When HIV-infected individuals are treated with combination antiretroviral therapy (cART), dramatic changes in the composition of the CD4^+^ and CD8^+^ T-cell pools are typically observed. We have previously shown that treatment with cART enables the CD4^+^ T-cell compartment to fully reconstitute in the majority of cases when HIV is successfully suppressed. CD4^+^ T-cell numbers increase gradually during the first years of cART to eventually reach age-matched control levels, and also the composition of the CD4^+^ T-cell pool becomes comparable to that of healthy, age-matched individuals (10). By contrast, HIV-induced changes in the CD8^+^ T-cell compartment seem to be more persistent. Increased CD8^+^ T-cell numbers have been reported even in patients on long-term (LT) cART and have been related to an increased risk of non-AIDS morbidity and mortality (11–14). The possible causes of these persistent changes remain debated and include residual viral load, residual immune activation, and CMV coinfection (12).

Interpreting alterations in the size and composition of the CD8^+^ T-cell pool is complicated by the influence of cytomegalovirus (CMV), a virus that is highly prevalent among healthy individuals and even more among HIV-infected individuals. The percentage of naïve CD8^+^ T-cells is typically lower and the percentage of effector CD8^+^ T-cells higher in CMV^+^ compared to CMV^−^ healthy individuals (15–20). Also, in terms of absolute cell numbers, memory and effector CD8^+^ T-cells tend to be more frequent in CMV^+^ compared to CMV^−^ healthy individuals (16–19, 21, 22). It is therefore important to carefully disentangle the influence of age and CMV (22, 23) when analyzing the changes in the CD8^+^ T-cell pools of LT-treated HIV-infected individuals.

Here, we studied the composition of the CD8^+^ T-cell compartment in LT cART-treated HIV-1-infected individuals who responded well to therapy, both in terms of control of virus load and CD4^+^ T-cell recovery and compared it to the changes in absolute numbers of naïve, central memory (CM), effector memory (EM), and effector CD8^+^ T-cells in CMV^+^ and CMV^−^ individuals during healthy aging. We found that most changes observed in the CD8^+^ T-cell pools of HIV-infected individuals on LT successful cART were a direct reflection of their CMV^+^ status. Only the LT enlargement of the CM CD8^+^ T-cell pool in LT-treated HIV patients could not be attributed directly to CMV.

## Materials and Methods

### Study Population

Thirty HIV-1-infected individuals of 18 years of age or older, who were under follow-up in the Department of Infectious Diseases of the University Medical Center Utrecht (UMCU Utrecht, The Netherlands), were included for cross-sectional analyses. At the moment of inclusion, they had been treated with cART for at least 7 years. In the last 5 years preceding study inclusion, they had undetectable HIV RNA plasma levels (<50 copies/ml) with no more than two isolated viral blips of HIV RNA (number of copies between 50 and 400/ml). Participants had to have CD4^+^ T-cell numbers above 500/µl of blood and express HLA-A2 and/or HLA-B8 alleles in order to measure their HIV-specific T-cell response using tetramers. Thirteen HIV-1-infected individuals (five from the original group and eight additional patients) were included in a longitudinal analysis. They were 18 years or older and had been treated with cART for at least 7 years, with undetectable HIV RNA plasma levels (<50 copies/ml) and CD4^+^ T-cell numbers above 400/µl of blood. In this group, a maximum of three viral blips above 50 copies/ml were allowed.

To study the effect of age and CMV in healthy individuals, we included 257 healthy donors, of which 119 adults (>18 years of age)—who were registered blood donors at the Dutch Blood Bank, or employees of the University Medical Center Utrecht—and 138 children (between 1 and 18 years of age) admitted to the UMCU to undergo elective urological, plastic, ophthalmologic or general surgery. To minimize interference on immunologic parameters, blood was drawn prior to or directly after the onset of anesthesia (24, 25). All participants were considered immunologically healthy, as they did not have any history of infectious diseases or hematological or immunological disorders, or showed any signs of acute infection at the time of venipuncture. CMV^+^ children had a mean age of 8 years and CMV^−^ children of 7 years. Both CMV^+^ and CMV^−^ adults had a mean age of 47 years. As a control group for the HIV-infected individuals, we used data from 107 (out of the 119) healthy adults so that the ages of the groups were matched. Basic characteristics of healthy and HIV-infected individuals are summarized in Table 1.

**Table 1:** Basic characteristics of healthy and HIV-infected individuals.

Healthy children	CMV^−^	CMV^+^
CMV status (%, number)	71%, *N* = 96	29%, *N* = 42
Age in years^a^	7 (1–18)	8 (1–17)
CD4^+^ T-cell count/µl blood at study moment^a^	1,538 (442–7,644)	1,318 (528–3,404)
CD8^+^ T-cell count/µl blood at study moment^a^	750 (158–3,586)	928 (265–2,417)
CD4/CD8 ratio at study moment^a^	2.1 (0.8–5.4)	1.6 (0.7–4.1)

**Healthy adults**	**CMV^−^**	**CMV^+^**

CMV status (%, number)	61%, *N* = 74	39%, *N* = 45
Age in years^a^	47 (20–70)	47 (23–69)
CD4^+^ T-cell count/µl blood at study moment^a^	1,741 (114–8,811)	1,739 (249–7,939)
CD8^+^ T-cell count/µl blood at study moment^a^	652 (95–3,856)	911 (125–6,943)
CD4/CD8 ratio at study moment^a^	2.9 (0.8–10)	2.3 (0.6–5.3)

**HIV-infected individuals with cross-sectional data only**		**Based on**

CD4^+^ T-cell count/µl blood at start cART^a^	282 (11–558)	*N* = 25
Viral load in copies/ml at start cART^a^	2.2 × 10^5^ (<400–7.5 × 10^5^)	*N* = 21
Years on cART^a^	11.1 (7.0–14.2)	*N* = 25
Age at LT^b^ cART in years^a^	48 (34–70)	*N* = 25
CD4^+^ T-cell count/µl blood at LT cART^a^	613 (214–1,173)	*N* = 25
CD8^+^ T-cell count/µl blood at LT cART^a^	750 (204–3,337)	*N* = 25
CD4/CD8 ratio at LT cART^a^	1.1 (0.1–4.2)	*N* = 25

**HIV-infected individuals with longitudinal data**		**Based on**

CD4^+^ T-cell count/µl blood at start cART^a^	275 (36–677)	*N* = 11
Viral load in copies/ml at start cART^a^	1.8 × 10^5^ (3.8.10^4^ to >5 × 10^5^)	*N* = 11
Years on cART^a^	8.1 (7.1–8.0)	*N* = 13
Age at LT cART in years^a^	47 (36–59)	*N* = 13
CD4^+^ T-cell count/µl blood at LT cART^a^	980 (500–1,470)	*N* = 13
CD8^+^ T-cell count/µl blood at LT cART^a^	800 (480–1,470)	*N* = 13
CD4/CD8 ratio at LT cART^a^	1.3 (0.5–2.2)	*N* = 13

*^a^Mean (range)*.

*^b^LT cART = long-term cART*.

All patients or their legal guardians gave written informed consent in agreement with the Declaration of Helsinki (version: 59th WMA General Assembly, Seoul, October 2008). The protocols were approved by the Medical Ethical Committee of the UMC Utrecht. Blood from healthy adult (>18 years of age) volunteers was obtained under guidelines of the Medical Ethical Committee of the UMC Utrecht or under guidelines of Sanquin (Blood Bank, The Netherlands).

### Flow Cytometry

Whole EDTA-anticoagulated or sodium heparine anticoagulated blood was obtained by venipuncture. PBMCs were obtained by Ficoll–Paque density gradient centrifugation directly analyzed or cryopreserved until further use. Absolute CD4^+^ and CD8^+^ T-cell numbers were determined by dual-platform flow cytometry, using TruCount tubes (BD Biosciences) or were calculated by multiplying the percentage of the indicated subset as obtained by flow cytometry and the absolute lymphocyte number as determined using a Cell-Dyn Sapphire hematology Analyzer (Abbott Diagnostics). Naïve (CD27^+^CD45RO^−^), CM (CD27^+^CD45RO^+^), EM (CD27^−^CD45RO^+^), and effector (CD27^−^ CD45RO^−^) CD8^+^ T-cells were assessed by flow cytometry. To this end, PBMCs were incubated with CD3 [PerCP or FITC (Biolegend)] or CD3-eFluor450 (eBioscience), CD8 [PerCP-Cy5.5, APC-Cy7, Amcyan or V500 (BD Biosciences)], CD27 [APC-Cy7 (BD Biosciences), APC, APC-AF750 (eBioscience) or FITC (Sanquin)] and CD45RO [PE or PE-Cy7 (BD Biosciences)] monoclonal antibodies (mAbs), in appropriate combinations. Within the subsets, characteristics of the cells were determined following standard staining protocols using CD28-FITC (BD Biosciences) and CD57-APC (Biolegend) mAbs for the level of senescence, AnnexinV-PE and 7AAD (BD Biosciences) mAbs for the level of apoptosis, and Ki67-FITC (DakoCytomation) mAbs as a measure of T-cell proliferation. HIV-specific CD8^+^ T-cells were detected using the following HLA-tetramers: HLA-B8-BFLKEKGGL, HLA-B8-EIYKRWII, and HLA-A2-SLYNTVATL, which were prepared as previously described (2). All experiments were analyzed on a FACS Canto II or FACS LSR II (BD Biosciences) with FACS Diva software (BD Biosciences).

### Measuring HIV Viral Load

Human immunodeficiency virus viral load monitoring was performed on EDTA plasma samples using the Roche Cobas Taqman v2.0 or the Roche Cobas Amplicor v1.5 assay. The result of the viral load determination was reported as a specified load (copies/ml), RNA detected, or target not detected (i.e. no signal in the PCR, no viremia) with cut-off values depending on the sensitivity of the two assays (i.e. >20, 0-20, 0 and >50, 0-50, 0 copies/ml, respectively).

### Analysis of CMV Serostatus

Analysis of CMV serostatus was performed in 30 HIV-infected individuals and 257 healthy individuals using the CMV IgG ELISA kit (IBL international) according to the manufacturer’s instructions, using plasma. Individuals who were CMV seropositive or seronegative according to this assay are referred to as CMV^+^ and CMV^−^, respectively, throughout the article.

### Statistical Analysis

Cross-sectional comparisons between HIV-infected individuals on LT cART and healthy individuals, and between CMV^+^ and CMV^−^ individuals were based on Mann–Whitney *U*-tests. Wilcoxon matched-pair signed rank tests were used for longitudinal analyses. CD8^+^ T-cell changes over the age of CMV^+^ and CMV^−^ individuals were studied using linear regression analysis; data from children and adults were analyzed separately. All statistical analyses were performed using the GraphPad Prism software (Graphpad Software, Inc.). To provide insight into the significance of observed differences, throughout the manuscript, we report exact *P*-values, without correction for multiple testing.

## Results

### Cell Numbers in Most CD8^+^ T-Cell Subsets Remain Elevated Despite LT Successful cART

To study to what extent the different CD8^+^ T-cell subsets normalized on LT cART, we measured the total CD8^+^ T-cell numbers and the number of cells in different CD8^+^ T-cell subsets of 38 HIV-infected individuals on LT successful cART and 107 healthy, age-matched controls. All HIV-1-infected individuals had a CD4^+^ T-cell count of >400 cells/μl at the time of inclusion and had undetectable viral loads for at least the last 5 years preceding study inclusion, with maximally two isolated blips (viral load between 50 and 400 copies/ml). The mean time on cART was 10 (range 7–14) years. The mean age of the 107 age-matched healthy donors was 50 (range 27–70) years, which was not significantly different from that of the HIV-infected individuals, which was 48 (range 34–70) years.

**Figure 1:**
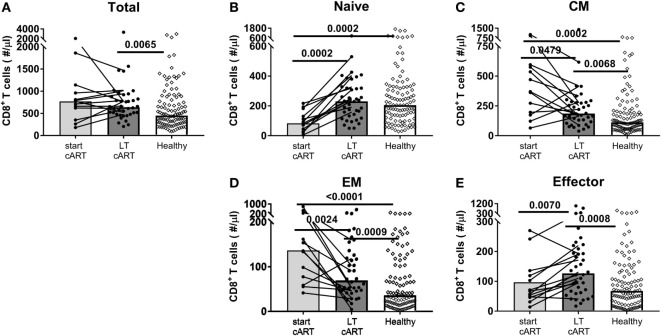
Potential of the CD8^+^ T-cell pool to normalize on long-term (LT) combination antiretroviral treatment (cART). **(A)** Total CD8^+^ T-cell numbers and **(B)** naïve (CD27^+^CD45RO^−^), **(C)** central memory (CM) (CD27^+^CD45RO^+^), **(D)** effector memory (EM) (CD27^−^CD45RO^+^), and **(E)** effector (CD27^−^CD45RO^+^) CD8^+^ T-cell numbers of human immunodeficiency virus (HIV)-infected individuals at a moment just before the start of cART (*N* = 13), during LT (i.e., at least 7 years of) cART (*N* = 38), and of [a mixed group of cytomegalovirus (CMV)^+^ and CMV^−^] age-matched healthy controls (*N* = 107). Bars represent median values of all individuals in a group, and data from the same individual are connected by lines. Comparisons between cross-sectional data from HIV-infected individuals and healthy individuals were based on a Mann–Whitney *U*-test. A Wilcoxon matched-pair signed rank test was used to study the significance of longitudinal changes in cell numbers from HIV-infected individuals. All (uncorrected) *P*-values of <0.05 are provided in the figure.

In line with what has previously been reported (11–14), the total CD8^+^ T-cell numbers remained significantly elevated (median 638 vs. 449 CD8^+^ T-cells/μl blood in HIV-infected and healthy individuals, respectively, Figure 1A) despite LT successful cART. Based on the expression of the markers CD27 and CD45RO, we distinguished between naïve (CD27^+^CD45RO^−^), CM (CD27^+^CD45RO^+^), EM (CD27^−^CD45RO^+^), and effector (CD27^−^CD45RO^−^) CD8^+^ T-cells. Naïve CD8^+^ T-cell numbers of HIV-infected individuals on LT cART (median 231 cells/μl blood) had normalized to levels comparable to healthy individuals (median 205 cells/μl blood, Figure 1B). By contrast, CM, EM, and effector CD8^+^ T-cell numbers remained nearly twofold increased compared to healthy age-matched individuals (Figures 1C–E).

### Memory CD8^+^ T-Cell Numbers Did Decline During cART

Since CM, EM, and effector CD8^+^ T-cell numbers had not normalized after at least 7 years of cART, we studied whether these subsets had been stably maintained during treatment or whether cART had decreased these cell numbers, albeit incompletely. To this end, we followed longitudinal changes in CD8^+^ T-cell numbers in 13 HIV-infected individuals, from pretreatment to at least 7 years of successful cART. Patients were selected to have CD4^+^ T-cell numbers of at least 400/μl blood. Two participants had one, one participant had two, and one participant had three occasional blips. Total CD8^+^ T-cell numbers remained relatively stable during LT treatment, at significantly higher levels than in healthy age-matched controls (Figure 1A). Nevertheless, there were substantial changes in the subset composition of the CD8^+^ T-cell pool on cART. Naïve CD8^+^ T-cell numbers, which were low at the start of cART, increased significantly over time and were no longer lower than in healthy controls (Figure 1B). By contrast, CM and EM CD8^+^ T-cell numbers decreased significantly during LT cART, but remained higher than in healthy controls, while effector CD8^+^ T-cell numbers showed a significant increase during LT treatment to significantly higher levels than in healthy controls (Figure 1B).

**Figure 2:**
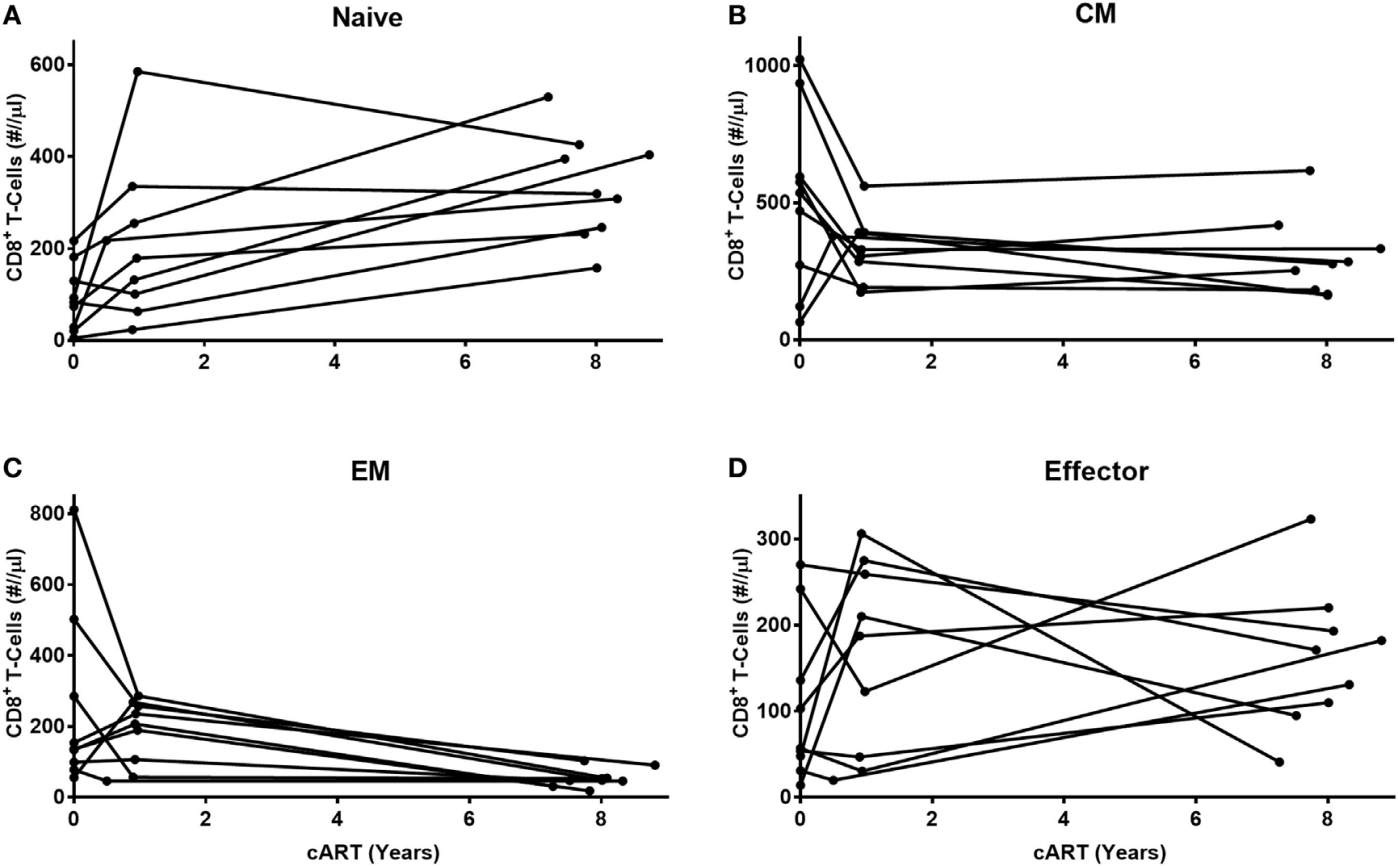
Early and late effects of combination anti-retroviral treatment (cART) on CD8^+^ T-cell numbers. Longitudinal analysis of cell numbers in four CD8^+^ T-cell subsets [**(A)** naïve, **(B)** central memory (CM), **(C)** effector memory (EM), and **(D)** effector] of nine HIV-infected individuals, during the first year of cART until at least 7 years of cART. Because of low sample sizes, we refrained from testing these changes statistically.

We next investigated whether the observed changes in the different CD8^+^ T-cell subsets during cART occurred gradually over time, or more abruptly at the start of cART. To this end, we compared CD8^+^ T-cell numbers pre-cART, 1 year after the start of cART and after LT cART in 9 of the 13 HIV-infected individuals (Figure 2). Naïve cell numbers changed quite gradually over time. By contrast, the dominant changes in CM and EM CD8^+^ T-cell numbers were observed during the first year of cART, after which CM numbers hardly changed and EM numbers declined more gradually. No clear pattern was observed for effector cells. Although we refrained from statistical analysis of these changes due to limited sample sizes, these data suggest that the successful suppression of HIV has a fast and large impact on memory CD8^+^ T-cell numbers.

### Changes in the CD8^+^ T-Cell Pool in CMV^+^ and CMV^−^ Healthy Individuals With Age

As CMV infection is known to increase CD8^+^ T-cell numbers in healthy individuals (16, 22), we studied whether the enlarged memory and effector CD8^+^ T-cell pools observed in LT-treated patients could be a direct reflection of the increased CMV prevalence among HIV-infected individuals. Indeed, 93% (*N* = 30) of HIV-infected individuals in our study had a detectable anti-CMV IgG titer (results not shown). To test this hypothesis, we first followed the changes in absolute numbers of naïve, CM, EM, and effector CD8^+^ T-cells in 87 CMV^+^ and 170 CMV^−^ healthy individuals of different ages, varying from 1 to 70 years.

**Figure 3:**
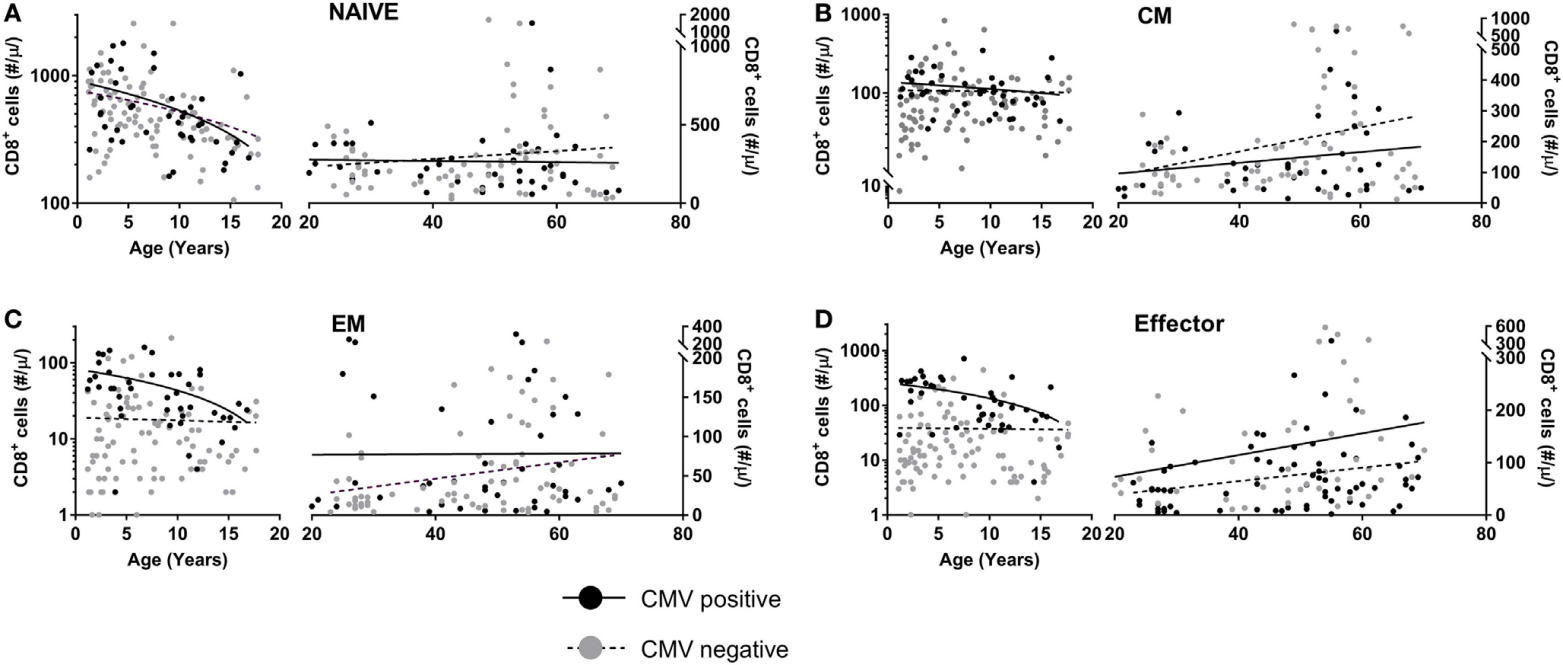
CD8^+^ T-cell numbers in cytomegalovirus (CMV)^+^ and CMV^−^ healthy individuals. Changes in absolute **(A)** naïve, **(B)** central memory (CM), **(C)** effector memory (EM), and **(D)** effector CD8^+^ T-cell numbers in CMV^+^ (black circles) and CMV^−^ (grey circles) healthy individuals with age. Solid curves represent linear regression lines through the data from adults and children, which were analyzed separately. Naïve cell numbers declined significantly with age in both CMV^+^ and CMV^−^ children, while EM and effector cell numbers declined significantly in CMV^+^ children only. CM, EM, and effector cell numbers increased significantly with age in CMV^−^ adults only.

Since T-cell numbers per ml blood in young children change considerably with age, possibly due to the growth of their blood volume (26), we separately analyzed the data obtained from children (<18 years of age) and adults (>18 years of age). In line with previous literature (22, 27), we observed a significant decline in naïve CD8^+^ T-cell numbers with age, both in CMV^+^ and in CMV^−^ individuals (Figure 3A), with no significant difference between their rates of decline. When limiting our analyses to adults, no further decline in naïve CD8^+^ T-cell numbers was observed. CM CD8^+^ T-cell numbers did not differ significantly between CMV^+^ and CMV^−^ healthy individuals and only showed a significant increase with age in CMV^−^ adults (Figure 3B). By contrast, EM and effector CD8^+^ T-cell numbers all increased significantly—albeit only mildly—with age in CMV^−^ adults but not in CMV^+^ adults (Figures 3C,D). When analyzed over the full age range, EM and effector CD8^+^ T-cell numbers were significantly elevated in CMV^+^ individuals, while naïve and CM CD8^+^ T-cell numbers were not. The fact that even in children, EM and effector CD8^+^ T-cell numbers were significantly higher in CMV^+^ compared to CMV^−^ individuals suggests that the effects of CMV on these subsets occur rapidly and do not require years to accumulate. Summarizing, the most significant differences in absolute CD8^+^ T-cell numbers between CMV^+^ and CMV^−^ healthy individuals were found in the EM and effector CD8^+^ T-cell compartments.

### CD8^+^ T-Cell Expansions During LT cART Are Largely Explained by CMV

**Figure 4:**
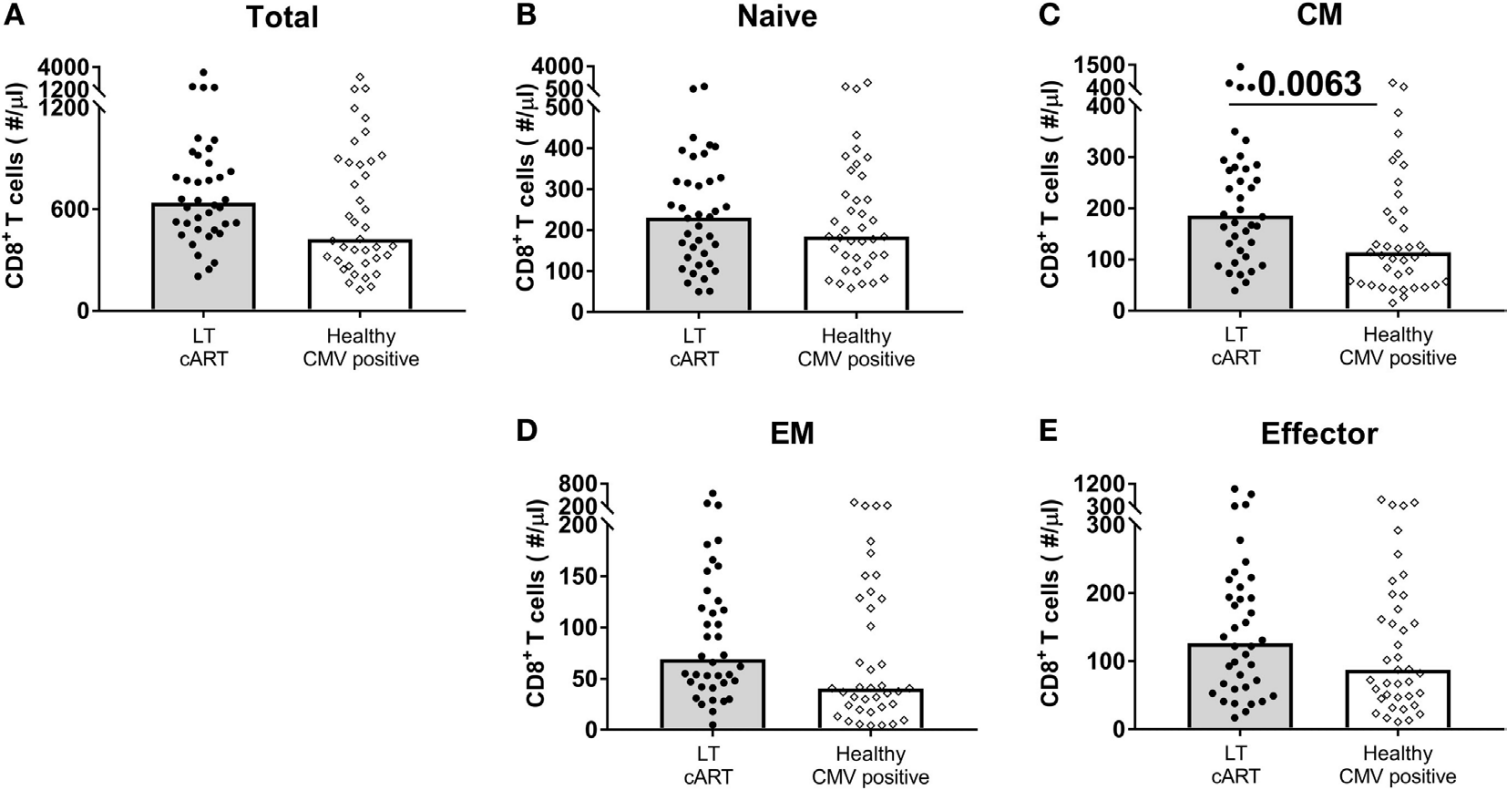
CD8^+^ T-cell numbers in cytomegalovirus (CMV)^+^ healthy individuals and human immunodeficiency virus (HIV) patients on long-term (LT) combination anti-retroviral treatment (cART). Comparison of **(A)** total, **(B)** naïve, **(C)** central memory (CM), **(D)** effector memory (EM), and **(E)** effector CD8^+^ T-cell numbers between HIV-infected individuals on LT cART (*N* = 38) and CMV^+^ age-matched healthy controls (*N* = 39). Bars represent median values. Comparisons between data from HIV-infected individuals and healthy individuals were based on a Mann–Whitney *U*-test. All (uncorrected) *P*-values of <0.05 are provided in the figure.

When comparing the sizes of the different CD8^+^ T-cell subsets in HIV-infected individuals on LT successful cART with those of CMV^+^ healthy age-matched individuals (*N* = 39), both EM and effector CD8^+^ T-cell numbers were no longer significantly increased (Figure 4). The relatively large sizes of the EM and effector CD8^+^ T-cell pools in LT-treated HIV-infected individuals may thus be a direct reflection of the increased prevalence of CMV among HIV-infected individuals. By contrast, CM CD8^+^ T-cell numbers were significantly higher (*P* = 0.0063) in LT-treated HIV patients, even when compared to those in CMV^+^ healthy age-matched controls. Their permanent increase despite years of successful cART could thus not be attributed directly to CMV.

### Elevated CM CD8^+^ T-Cell Numbers Are Explained Neither by Low-Level Viremia Nor by HIV-Specific T-Cells

**Figure 5:**
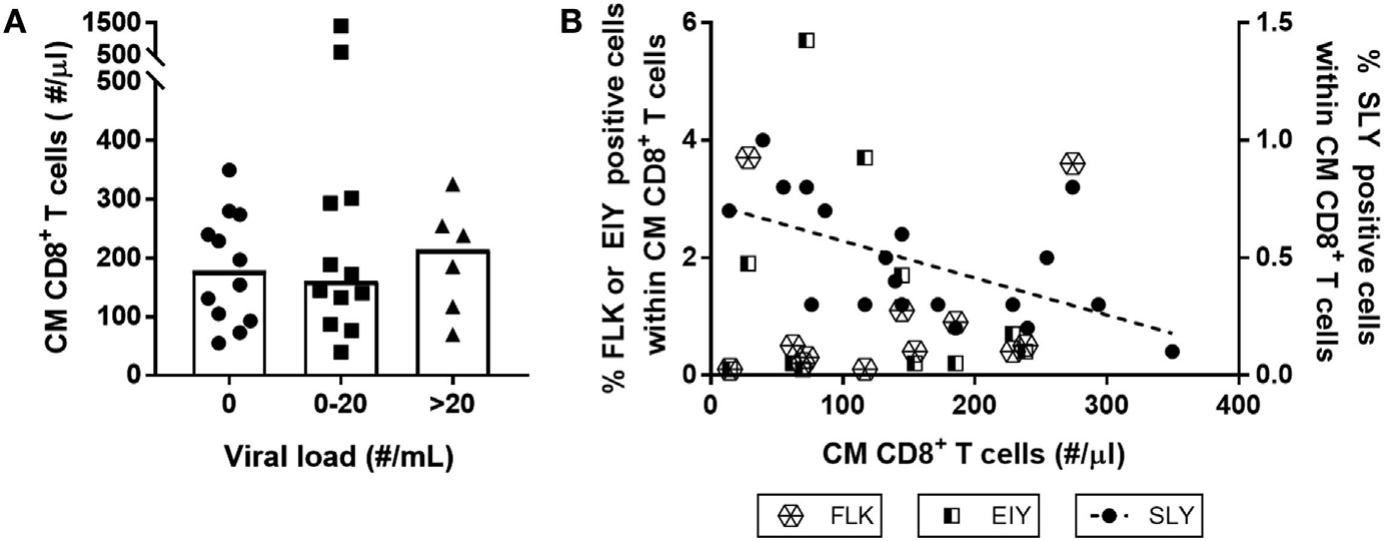
Neither low-level human immunodeficiency virus (HIV) viremia nor the presence of HIV-specific T-cells explain increased central memory (CM) CD8^+^ T-cell numbers during long-term (LT) combination anti-retroviral treatment (cART). CM CD8^+^ T-cell numbers in HIV-infected individuals on LT cART were neither associated with **(A)** levels of plasma HIV RNA load (grouped by the number of viral RNA copies/ml plasma determined using the Roche Cobas Taqman v2.0 assay), nor by **(B)** the percentage of HIV-specific CM CD8^+^ T-cells, measured using tetramers of the three immuno-dominant HIV-1 peptides: HLA-A2-SLYNTVATL (SLY), HLA-B8-FLKEKGGL (FLK), and HLA-B8-EIYKRWII (EIY). Depicted are individual values (symbols), medians (bars in panel A), and a linear regression line for the results of HLA-A2-SLYNTVATL (*P* = 0.01).

We next investigated whether low-level HIV viremia could explain why the CM CD8^+^ T-cell pool of LT-treated patients failed to normalize. Although all HIV-infected individuals had HIV RNA plasma loads below 50 copies/ml at the time of inclusion in this study, we could not exclude the possibility that differences in viremia below 50 copies/ml caused the CM CD8^+^ T-cell pool to remain expanded. We therefore tested whether HIV plasma load below the commonly used detection limit of 50 HIV RNA copies/ml plasma correlated with the number of CM CD8^+^ T-cells after LT cART. We found plasma levels above 20 copies/ml in 20% and between 0 and 20 copies/ml in 40% of the HIV-infected individuals; nevertheless, individuals having these increased plasma levels did not have significantly higher CM CD8^+^ T-cell numbers after LT cART than individuals who had no detectable HIV load (Figure 5A).

Alternatively, increased CM CD8^+^ T-cell numbers on LT cART may reflect the presence/persistence of HIV-specific memory CD8^+^ T-cells. We studied whether the presence of such responses correlated with absolute CM CD8^+^ T-cell numbers in patients on LT cART. To this end, we used tetramers of three immune-dominant HIV-1 peptides—the HLA-A2 restricted SLYNTVATL peptide and the HLA-B8 restricted FLKEKGGL and EIYKRWII peptides—to measure the frequencies of antigen-specific T-cells in HLA-A2 and/or HLA-B8 expressing individuals. We found no evidence for a positive correlation between the frequencies of HIV-specific CM CD8^+^ T-cell responses and numbers of CM CD8^+^ T-cells in LT-treated patients. The response to the HLA-A2 restricted SLYNTVATL peptide was even negatively correlated (*P* = 0.01) with the number of CM CD8^+^ T-cells (Figure 5B). Although HIV replication has been shown to be strongly correlated with increased HIV-specific CD8^+^ T-cell numbers (2, 28–30), we found no significant association between HIV plasma levels and frequencies of HIV-specific CM CD8^+^ T-cells (data not shown).

Taken together, these results suggest that neither HIV RNA plasma levels nor the LT maintenance of HIV-specific memory CD8^+^ T-cells could explain the lasting expansion of the CM CD8^+^ T-cell subset in LT-treated patients.

### Dynamic Properties of CD8^+^ T-Cells in CMV and After LT cART

**Figure 6:**
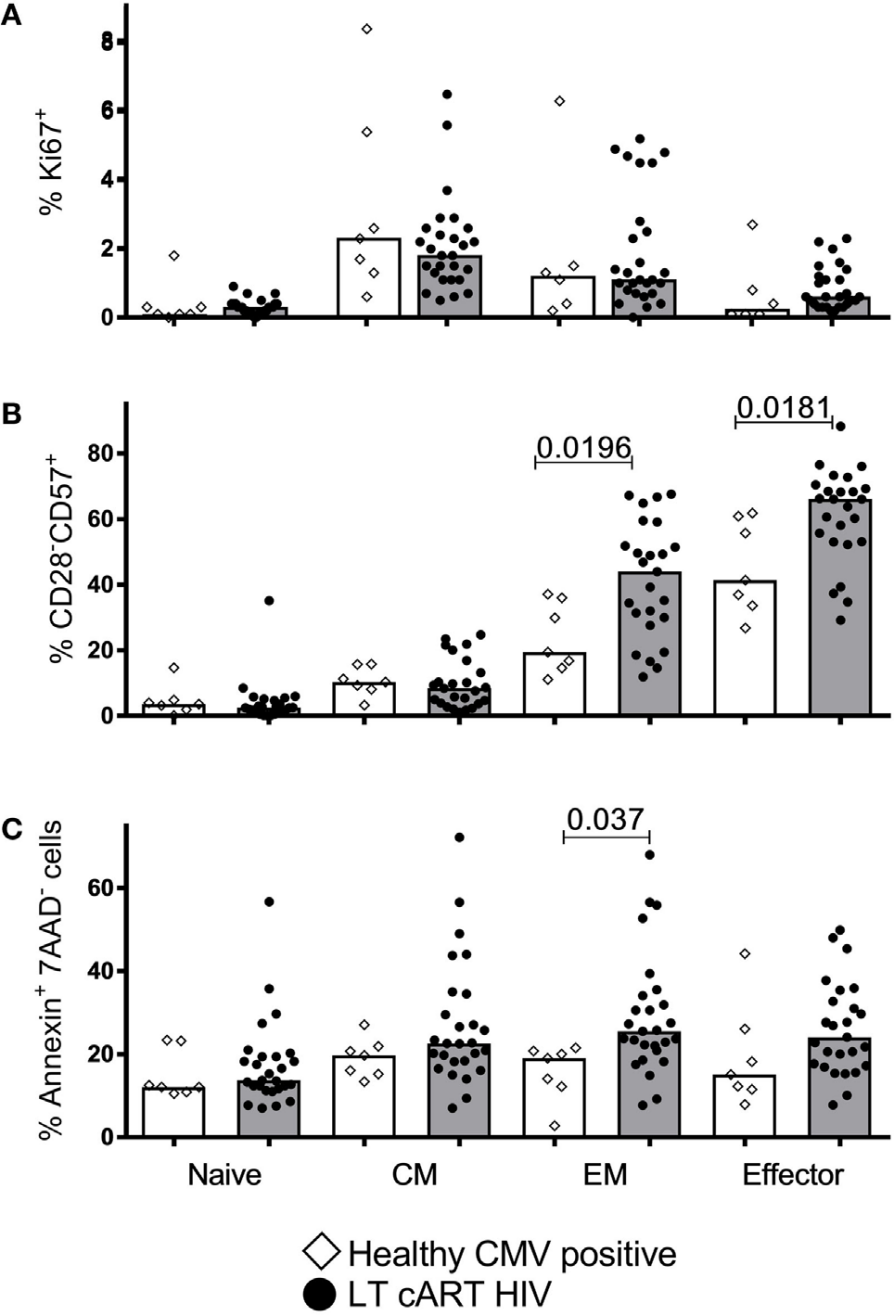
T-cell proliferation, senescence, and apoptosis. Percentages of **(A)** proliferating (Ki67^+^), **(B)** senescent (CD28^−^CD57^+^), and **(C)** apoptotic (AnnexinV^+^7AAD^−^) CD8^+^ T-cells in the different subsets in human immunodeficiency virus (HIV)-infected individuals on long-term combination anti-retroviral treatment (cART) (filled circles) compared to cytomegalovirus (CMV)^+^ healthy age-matched controls (open diamonds). Bars depict medians. Comparisons between data from HIV-infected individuals and healthy individuals were based on a Mann–Whitney *U*-test. All (uncorrected) *P*-values of <0.05 are provided in the figure.

Finally, we investigated to what extent LT cART led to normalization of cellular dynamics, including the fraction of proliferating (Ki67^+^), senescent (CD28^−^CD57^+^), and apoptotic (AnnexinV^+^7AAD^−^) cells. For CM cells, we found no significant differences in these characteristics between HIV patients on LT successful cART and CMV^+^ healthy controls (Figure 6). The fraction of apoptotic EM cells and the fraction of senescent EM and effector cells were significantly increased in LT-treated patients, although the significance of these differences would be lost when correcting for multiple testing (e.g., using a Bonferroni correction). Taken together, we conclude that the increased CM CD8^+^ T-cell numbers in HIV-infected individuals on LT cART could not be explained by maintenance through an increased proliferation or resistance to apoptosis. Normalization of cell numbers in the other subsets tended to coincide with normalization of cell dynamics, although the percentage of senescent cells in the EM and effector subsets may be increased in LT cART-treated patients.

## Discussion

Changes in the CD8^+^ T-cell compartment of HIV-infected individuals are often compared to changes that occur during chronological aging, and HIV infection has therefore been described as a condition of accelerated immunological aging. In line with previous findings, we found that CD8^+^ T-cell numbers in LT-treated HIV patients remained significantly increased compared to (a mixture of CMV^+^ and CMV^−^) healthy age-matched controls, despite years of successful treatment. These persistent changes pertained to the CM, EM, and effector cell subsets, while the number of naïve CD8^+^ T-cells normalized to healthy age-matched levels. Our results suggest that the persistent expansions in the CD8^+^ T-cell pool of cART-treated individuals were a direct reflection of the increased prevalence of CMV among HIV-infected individuals. Indeed, the sizes of the EM and effector CD8^+^ T-cell pools of HIV patients on LT cART did not differ significantly from those of CMV^+^ healthy age-matched controls. We therefore conclude that the CD8^+^ T-cell pool of HIV-infected individuals—just like the CD4^+^ T-cell pool (10)—has the potential to normalize to a great extent on LT cART. Importantly, however, this normalization only becomes apparent when comparing to a healthy, age-matched, and CMV status-matched control group. Similarly, it was recently shown that the percentage of terminally differentiated T-cells in patients on LT cART was significantly higher than in unselected healthy blood bank donors, but similar to non-infected individuals matched for lifestyle and demographic factors with a higher prevalence of CMV (20). In our data, only the increased size of the CM CD8^+^ T-cell pool in HIV patients could not be explained directly by CMV.

Naïve CD8^+^ T-cell numbers tend to increase gradually during cART, and it was previously reported that 1.5 years of treatment is insufficient for complete normalization of the naïve CD8^+^ T-cell pool (29). We here found that 7 years of successful cART was sufficient for normalization of naïve CD8^+^ T-cell numbers, probably through a combination of low-level T-cell proliferation and *de novo* T-cell production by the thymus. In contrast to the gradual increase in cell numbers that we observed for naïve CD8^+^ T-cells, EM and CM CD8^+^ T-cell numbers underwent the largest changes during the first year of cART, after which cell numbers declined much more gradually or even remained constant. A similar biphasic pattern was observed for total CD8^+^ T-cell counts in a large-scale study among treated HIV-infected individuals (13). These changes match the changes in immune activation levels that are typically observed during cART, with a major decline in immune activation upon the initiation of cART and much more subtle changes in later years of treatment (31). Of the four CD8^+^ T-cell populations investigated, the effector population was the only population that increased during cART to levels higher than in healthy age-matched controls. A similar gradual accumulation of highly differentiated effector T-cells has been observed in healthy aging (32), as well as in untreated HIV infection (1). In accordance with the skewing of HIV-specific CD8^+^ T-cells toward a CM phenotype (3, 33), we found hardly any HIV-specific CD8^+^ T-cells in the effector compartment when staining with HIV tetramers (data not shown). The increased cell numbers in the effector compartment are thus not likely explained by the accumulation of HIV-specific T-cells. It was previously shown that the frequency of CMV-specific effector T-cells in HIV-infected individuals on cART (with undetectable viral load) was higher than in age-matched untreated HIV-infected individuals or healthy age-matched controls and was in fact comparable to that in the elderly (34). Since the prevalence of CMV in HIV-infected individuals was nearly 100%, it is plausible that infection with CMV is the driving force behind the increase in effector CD8^+^ T-cell numbers during cART, as it is in healthy individuals (16). The change that is perhaps least well understood is the persistent expansion of the CM CD8^+^ T-cell pool in patients on cART. Consistent with earlier findings on total CD8^+^ T-cell counts in treated HIV patients (13), increased CM T-cell numbers were neither related to residual HIV plasma load nor to the presence of HIV-specific T-cells. We also found no indications for increased levels of proliferation or apoptosis resistance of these cells.

We here show that also in terms of proliferation, senescence, and apoptosis, the CD8^+^ T-cell pool of HIV-infected individuals on LT successful cART tends to normalize to levels observed in CMV^+^ healthy age-matched controls, perhaps with the exception of increased senescence of EM and effector CD8^+^ T-cells. In a previous deuterium-labeling study in HIV-infected individuals who had been successfully treated with cART for at least 1 year, we observed that the turnover of the memory T-cell populations had already nearly normalized, while the turnover of naïve CD4^+^ and CD8^+^ T-cells had not yet normalized (35). Perhaps, it is not surprising that the naïve T-cell pool, which normalized most gradually in terms of cell numbers, also took more time to normalize in terms of cellular turnover. An earlier paper by Wittkop et al. (36) reported significantly increased levels of CD8^+^ T-cell activation after 5 years of cART. However, in contrast to our study, the study performed by Wittkop et al. (36) was not restricted to immunological responders, which might explain the discrepancy and suggests that in immunological non-responders, immune activation may persist.

In support of our interpretation that the increased EM and effector CD8^+^ T-cell numbers in patients on LT cART may be a direct reflection of the CMV^+^ status of these individuals, a previous study showed that CD8^+^ T-cell numbers in HIV patients on LT cART were significantly increased in CMV^+^ but not in CMV^−^ individuals (37). In line with this, CD4/CD8 T-cell ratios were found to be significantly higher in CMV^+^ compared to CMV^−^ cART-treated individuals with good CD4^+^ T-cell reconstitution (38). In our cohort, only 2 out of 30 HIV-infected individuals were CMV^−^, which hampered a direct comparison between CMV^+^ and CMV^−^ HIV-infected individuals.

It has previously been reported that both age and CMV have a significant effect on CD8^+^ T-cell numbers (16, 22). In line with previous literature (16–19, 22, 39), EM and effector CD8^+^ T-cell numbers were significantly higher in CMV^+^ compared to CMV^−^ healthy individuals. This expansion may for a large part be composed of CMV-specific T-cells, since CD8^+^ T-cells specific for the major immediate early 1 protein (IE-1) or the structural phosphoprotein pp65 have been described to occupy up to 8% of the total CD8^+^ T-cell pool in adults (34, 40). Based on the combined responses against IE-1, pp65, and nonstructural phosphoprotein pp50, it has been estimated that up to 45% of total CD8^+^ T-cells may be CMV-specific in the elderly (41). In line with previous data showing that CMV-specific CD8^+^ T-cells reside mainly in the effector CD8^+^ T-cell pool and to a lesser extent in the EM subset (3), we observed a larger expansion of effector CD8^+^ T-cell numbers compared to EM CD8^+^ T-cell numbers. Interestingly, EM and effector CD8^+^ T-cell numbers in very young CMV^+^ individuals were also significantly elevated, suggesting that cell numbers in these subsets increase quickly after CMV infection and do not take years to accumulate.

A previous report by Wertheimer et al. (22), which beautifully disentangled the effects of age and CMV in a large cohort of healthy individuals, reported no significant increase in EM and CM CD8^+^ T-cell numbers with age in CMV^−^ adults. We did observe a significant—albeit mild—increase in these subsets with age in CMV^−^ adults. In line with Wertheimer et al. (22), we found no significant difference in naïve CD8^+^ T-cell numbers between CMV^+^ and CMV^−^ adults. By contrast, a significant reduction in absolute naïve CD8^+^ T-cell numbers (based on the expression of LFA-1 and CD45RA) in CMV^+^ compared to CMV^−^ adults was previously reported (16). These seemingly conflicting results may be due to the fact that inter-individual differences in T-cell counts tend to be larger than the effect of age on memory T-cell counts and the effect of CMV on naïve T-cell counts, respectively.

While our data suggest that most changes in the CD8^+^ T-cell pool of cART-treated individuals are a direct and natural result of CMV—as also observed in healthy CMV^+^ individuals—another study suggested that it is the *combination* of HIV and CMV that drives persistent CD8^+^ T-cell expansions (37). Indeed, increased CD8^+^ T-cell numbers in the latter study were only found in CMV^+^ and not in CMV^−^ cART-treated patients. Whether directly or *via* interaction with HIV, both Freeman et al. (2016) (37) and our data suggest that CMV is a central player in the persistent changes in the CD8^+^ T-cell pool during cART. We can nevertheless not exclude the possibility that other factors play a role. In fact, our own comparison of CD8^+^ T-cell numbers in cART-treated and CMV^+^ healthy individuals (Figure 4) suggests that the expansions in cART may be larger (although not significantly) than in CMV^+^ healthy individuals. Residual immune activation, which itself may be related to CMV (37), may be another driver for CD8^+^ T-cell expansions in (treated) HIV (12, 38).

Taken together, our findings show that the CD8^+^ T-cell pool has great potential to normalize in HIV-infected individuals who respond well to treatment and underline the importance of matching not only for age but also for CMV serostatus (20). In this light, also previous interpretations of changes in the composition or dynamics of the T-cell pool in HIV-infected individuals may have to be reconsidered if they did not take the effects of CMV into consideration.

## Ethics Statement

All patients or their legal guardians gave written informed consent in agreement with the Declaration of Helsinki (version: 59th WMA General Assembly, Seoul, October 2008). The protocols were approved by the Medical Ethical Committee of the University Medical Center (UMC) Utrecht. Blood from healthy adult (>18 years of age) volunteers was obtained under guidelines of the Medical Ethical Committee of the UMC Utrecht or under guidelines of Sanguin (Blood Bank, The Netherlands).

## Author Contributions

EV, JB, and KT designed the work. EV, LW, RG, BR, HR, LB, and SO acquired and analyzed the immunological data. AW acquired and analyzed the virological data. AH and TM analyzed patient characteristics, selected and included patients. EV, JB, and KT drafted the manuscript and all authors critically revised the intellectual content of the manuscript.

## Conflict of Interest Statement

The authors declare that the research was conducted in the absence of any commercial or financial relationships that could be construed as a potential conflict of interest.
